# Gonadal, Not Maternal, Acquisition of Duplicated *pax6* Orthologs in *Megalobrama Amblycephala*

**DOI:** 10.3390/ijms20071710

**Published:** 2019-04-05

**Authors:** Qihua Pan, Ting Xue, Bilin Xia, Junzhi Luo, Qian Wang, Yuewen Jiang, Miao Yu, Tiansheng Chen

**Affiliations:** 1 Key Laboratory of Freshwater Animal Breeding, Ministry of Agriculture and College of Fisheries, Huazhong Agricultural University, Wuhan 430070, China; panqihua@webmail.hzau.edu.cn (Q.P.); xue2011ting124@126.com (T.X.); xblin@webmail.hzau.edu.cn (B.X.); luojunzhi3216@163.com (J.L.); qian.wang@webmail.hzau.edu.cn (Q.W.); 13477032785@163.com (Y.J.); miaoyu@htu.edu.cn (M.Y.); 2 College of Fishery, Engineering Technology Research Center of Henan Province for Aquatic Animal Cultivation, Henan Normal University, Xinxiang 453007, China; 3 Collaborative Innovation Center for Efficient and Health Production of Fisheries in Hunan Province, Changde 41500, China

**Keywords:** blunt-snout bream (*Megalobrama amblycephala*), *pax6*, duplication, gonadal, maternal, expression

## Abstract

The highly conserved transcription factor Pax6 is involved in the development of the eyes, brain, and pancreas in vertebrates and invertebrates, whereas the additional expression pattern in other organs is still elusive. In this study, we cloned and characterized two *pax6* homologs in blunt snout bream (*Megalobrama amblycephala*), named *Mapax6a* and *Mapax6b*. The protein alignment and phylogenetic tree showed that Mapax6a and Mapax6b were highly conserved compared with their counterparts in other species. Genomic information analysis revealed that the synteny conservation of Wilms tumor, Aniridia, genitourinary abnormalities, and mental retardation loci was also maintained in this species. By reverse transcription polymerase chain reaction, the expression of *Mapax6a* was later than that of *Mapax6b* which was found in the blastula stage, while the expression of *Mapax6a* started from the somite stage, and both of them persisted in a subsequent stage during the embryonic development. By RNA and protein detection, *Mapax6a* and *Mapax6b* were detected in the eye and brain as canonic patterns, and most importantly, they were also enriched in germ cells of the testis and ovary. Therefore, our findings validate the duplication of *pax6* in fish, confirm the classical expression patterns in the brain and eye, and, for the first time, present a new acquisition of *Mapax6a* and *Mapax6b* in gonadal germ cells in particular. Therefore, our results enrich the expression pattern and evolutionary relationship of *pax6* by suggesting that duplicated *Mapax6* is involved in gametogenesis in *Megalobrama amblycephala*.

## 1. Introduction

*Paired box* (*PAX*) gene family proteins are an important transcription factor in vertebrates and invertebrates and include nine members, named *Pax1* to *Pax9* based on the protein domains [1]. As one of the members of the *PAX* gene family, *Pax6* is involved in cell differentiation, migration, and proliferation [2,3,4]. Previous studies have shown that *Pax6* plays an essential role in the organic development of various species such as eye, brain, and pancreas. Deletion or mutation of the *PAX6* gene results in ectopic eyes in human [5], mouse [6], zebrafish [7], and fruit fly [5,6,7,8]. In the development of the brain, *Pax6* likely involves p53 to regulate neuronal survival [9]. Other investigations have indicated that *Pax6* is necessary for the differentiation of glucagon-producing α-cells and transactivates the insulin promoters in the pancreas [10,11]. Expression of *pax6* in the gonad has been barely mentioned. The expression of *pax6* was found in the hermaphroditic gonads of *Dicyemid mesozoans*, although it was weak [12]. Subsequently, *Pax6* was detected in mouse spermatogenesis including spermatogonia, spermatocytes, and round spermatids [13]. Furthermore, the transcription factor *vab-3*/*pax6* was demonstrated to control the *Caenorhabditis elegans* hermaphrodite gonad size and shape by regulating the α integrin genes [14]. These results suggested that *Pax6* probably also affects sexual reproduction and fertility in animal phyla.

The teleost genome was duplicated based on vertebrate evolution, and the released genome data showed more genes producing functional divergence such as sub-functionalization or non-functionalization, even neofunctionalization [15,16]. In the literature, pax6 was shown to be duplicated in medaka [17], zebrafish [7,18,19], and elephant shark [20]. The zebrafish *pax6b* resembles the phenotypically heterozygous *Pax6* mutant mice and humans, and *pax6b* and *pax6a* shared the sub-functionalization in zebrafish [19,21]. In contrast, only one *pax6* was identified in Fugu [22]. Consequently, the duplicated *pax6* may have divergent expression patterns or even function in different teleosts.

Blunt snout bream (*Megalobrama amblycephala*) is known as one of the main freshwater aquaculture species in China, belonging to Cyprinomorpha, Cyprinidae, and *Megalobrama*. The study of blunt snout bream has advanced in the past decade, including genetics [23], nutrition [24], cryobiology [25], disease and immunology [26], and stem cells [27]. Notably, the released genomic data and identification of the transcription factors involved in gonadal development provide a molecular foundation for artificial breeding [28]. In the present study, we identify two *pax6* homologs, namely, *Mapax6a* and *Mapax6b*, in *Megalobrama amblycephala*. Subsequently, we describe the expression patterns of *Mapax6a* and *Mapax6b* during gametogenesis and embryogenesis.

## 2. Results 

### 2.1. Cloning of Mapax6a and Mapax6b 

**Figure 1:**
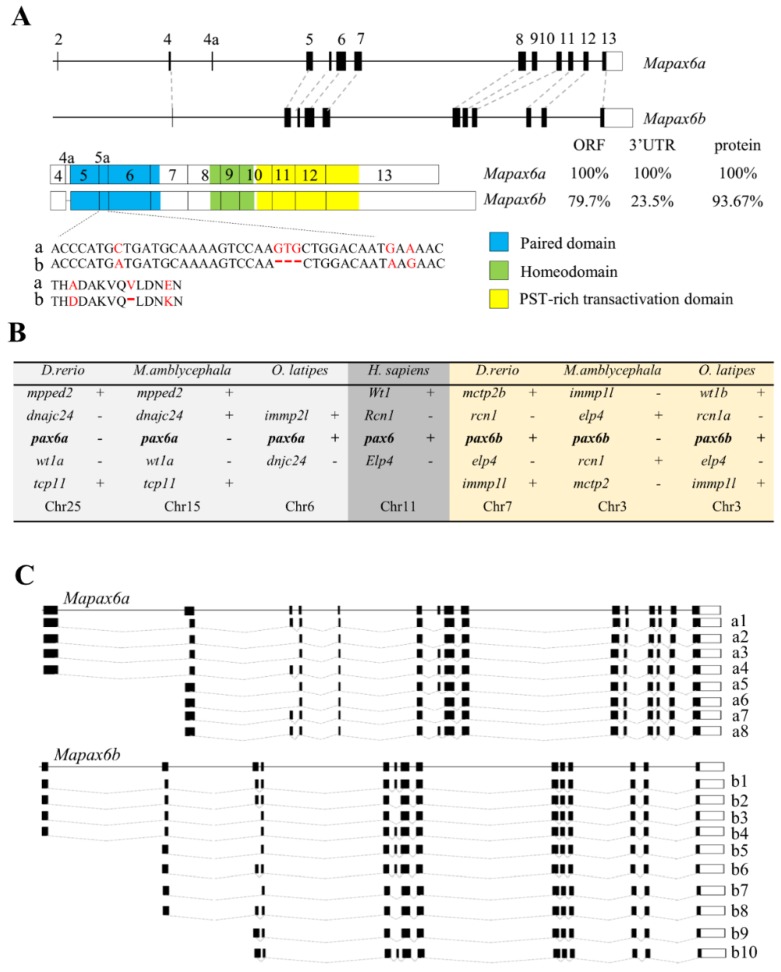
Comparison of the two *pax6* genes of *Megalobrama amblycephala*. (**A**) The genomic structures of *Mapax6a* and *Mapax6b* are shown. Conserved exons were connected by dotted lines. Both the translation-start sites (ATG) of *Mapax6a* and *Mapax6b* are located in exon 4. The mRNA structure is under the genomic structure. The paired domain is stained in blue, the green area is the homeodomain, and the yellow represents the proline–serine–threonine (PST)-rich transactivation domain. The comparison percentages of the open reading frame (ORF), 3′ untranslated region (UTR), and protein between *Mapax6a* and *Mapax6b* were analyzed by DNAMAN software. An alignment of the nucleic acid and amino acid sequence of exon 5a is under the mRNA structure (a/b). (**B**). Synteny conservation of pax6 and adjacent genes in different species. The duplicated pax6s are indicated in medaka and zebrafish. +: forward direction; -: reverse direction. (**C**) Summary isoforms of *Mapax6a* and *Mapax6b*. RNA-seq detected all of these isoforms, which were produced from alternative exons such as exon 2, exon3, and exon 5a.

According to an unpublished transcriptome database, we amplified the partial cDNA of putative *Mapax6a* and *Mapax6b*, including the open reading frame (ORF) and 3′ untranslated region (UTR), by RT-PCR and rapid amplification of cDNA ends (RACE). The length of *Mapax6a* (GenBank accession number: MF421242) is 1720 bp, and that of *Mapax6b* (GenBank accession number: MF421243) is 2128 bp (Supplementary Figure S1). Sequence analysis showed that *Mapax6a* contains 1326 bp of ORF coding 442 amino acids, and *Mapax6b* comprises 432 amino acids coded by the 1296 bp ORF. The difference in ORF length between *Mapax6a* and *Mapax6b* is due to lacking exon 4a in *Mapax6b*. The length of the *Mapax6a* 3′UTR is 389 bp, while the length of the *Mapax6b* 3′UTR is 828 bp. By a comparison of sequences of the ORF and 3′UTR, the identities are 79.7% in the ORF and 23.5% in the 3′UTR (Figure 1A; Supplementary Figure S2A,B), suggesting the conservation of the ORF between *Mapax6a* and *Mapax6b*. The alignment of deduced amino acids showed 93.67% identities between Mapax6a and Mapax6b (Figure 1A; Supplementary Figure S2C). We performed basic local alignment search tool (BLAST) with the two sequences in the available genome database [23], in which the *Mapax6a* locates in chromosome 15 and *Mapax6b* locates in chromosome 3 (Figure 1B). The *Mapax6a* has 15 exons, including an additional 14 amino acid residues (THADAKVQVLDNEN) encoded by exon 5a, whereas the *Mapax6b* contains 14 exons and also has an additional 13 amino acids (THDDAKVQLDNKN) encoded by exon 5a, and the insertion of exon 5a disrupts the DNA-binding properties of the paired domain (Figure 1A). Interestingly, exon 4a was not detected by sequence alignment in the *Mapax6b* genomic DNA (Figure 1A). Based on the transcriptome data and PCR amplification, *Mapax6a* contains 8 isoforms and *Mapax6b* has 10 isoforms, and the generation of isoforms resulted from the alternative splicing of exons such as exon 2, exon 3, and exon 5a (Figure 1C; Supplementary Figure S3). A cross-species comparison of a chromosomal location indicated that the *Mapax6* contained region has conserved synteny to the zebrafish *pax6* (*Drpax6*) and medaka *pax6 (Olpax6)* contained regions. Furthermore, the homologs of these genes near *pax6* were located in the WAGR (Wilms tumor, Aniridia, genitourinary abnormalities, and mental retardation) region of human (Figure 1B). Thus, *Mapax6* shows good syntenic conservation with other species.

### 2.2. Molecular Characterization of *Mapax6a* and *Mapax6b*

**Figure 2:**
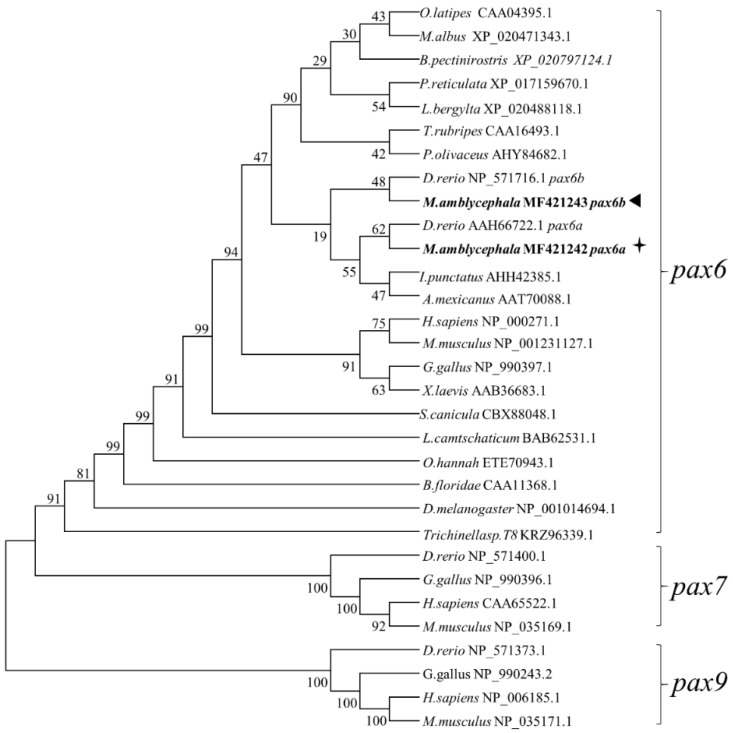
Molecular phylogenetic analysis of *pax6* homologs. The phylogenetic relationship of PAX family proteins was analyzed using the MEGA6 program by bootstrap analysis using neighbor-joining (1000 replicates). The numbers at the forks are the bootstrap proportions for each branch. A star indicates Mapax6a, and a triangle indicates Mapax6b.

By synteny analysis of the fish *pax6* in zebrafish, medaka, and human, *pax6* was duplicated in fish rather than in mammals. To further explore the relationship of *Mapax6a* and *Mapax6b* with other PAX families, their protein sequences were aligned using Vector NTI software. The identity analysis showed that Mapax6a and Mapax6b are highly conservative when compared with other species, from 89.66% to 95.12% (Supplementary Figure S4). Similar with other vertebrates, the predicted proteins of Mapax6a and Mapax6b also contain three conserved domains, including a pair domain (PD), a homeodomain (HD), and a proline–serine–threonine (PST)-rich transactivation region (Figure 1A; Supplementary Figure S3). The classic isoform raised by exon 5a in mammals was also identified in Mapax6a and Mapax6b. Constructed using MEGA 6.0 with the neighbor-joining method, the phylogenetic tree suggested the phylogenetic relationship among Mapax6a, Mapax6b, and other species’ pax6 proteins (Figure 2). The result showed that Mapax6a and Mapax6b are clustered with pax6 homologs from vertebrates and invertebrates and separated from other PAX family proteins such as pax7 and pax9 homologs. Clustered with zebrafish pax6a and pax6b together, Mapax6a and Mapax6b were therefore demonstrated to be pax6 homologs.

### 2.3. Different Expression Patterns between Mapax6a and Mapax6b 

**Figure 3:**
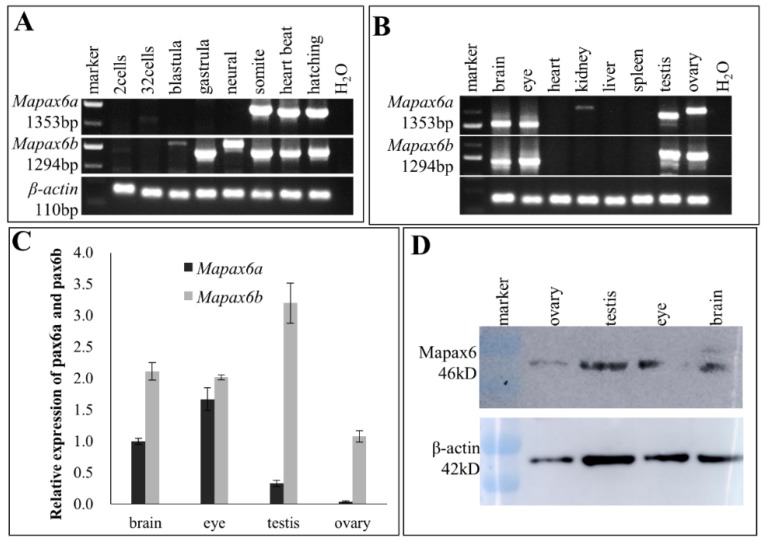
The expression patterns of *Mapax6a* and *Mapax6b*. (**A**) *Mapax6a* and *Mapax6b* expression in developing embryos. *Mapax6a* began to express at the somite stage, and *Mapax6b* was detected in the gastrula stage—earlier than *Mapax6a*. (**B**) *Mapax6a* and *Mapax6b* expression in adult tissues. *Mapax6a* and *Mapax6b* were both detected in the brain, eye, testis, and ovary. (**C**) Quantitative RT-PCR analysis of *Mapax6a* and *Mapax6b* in the brain, eye, testis, and ovary. Black bars represent *Mapax6a*, and gray bars represent *Mapax6b*. *β-actin* RNA was an internal reference. (**D**) Mapax6 protein detection by Western blot in different tissues. β-actin protein was an internal reference.

For the expression analysis, the full-length transcripts of *Mapax6a* and *Mapax6b* were examined by Semi-Quantitative Reverse Transcription Polymerase Chain Reaction (sqRT-PCR) during embryonic development. Unexpectedly, the transcripts of *Mapax6a* were found in the somite stage, heart-beat stage, and hatching, whereas the RNA expression of *Mapax6b* started in the blastula stage and was present in the subsequent stage (Figure 3A). Similarly, sqRT-PCR was used to detect the transcripts of *Mapax6a* and *Mapax6b* in adult tissues. The result revealed that both of them were found in the brain and eye (Figure 3B,C). Interestingly, they also were detected in the testis and ovary (Figure 3B,C), and the presence of the pax6 protein was also validated in the brain, eye, testis, and ovary by Western blot (Figure 3D). 

**Figure 4:**
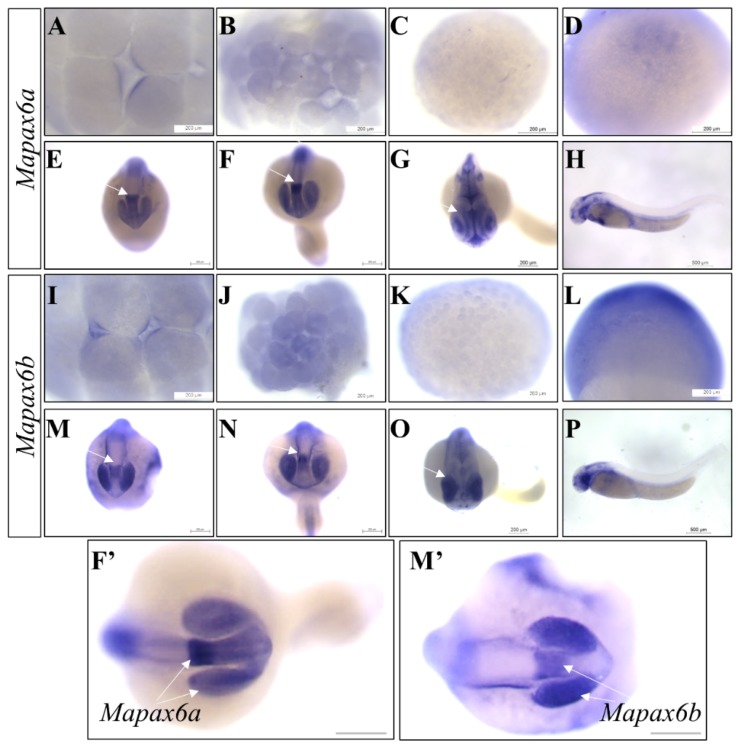
RNA expression of *Mapax6a* and *Mapax6b* during embryogenesis. Whole embryos at various stages were subjected to in situ hybridization (ISH) with antisense riboprobes of *Mapax6a* and *Mapax6b* and observed under microscopy. (**A–H**) The signals were stained with *Mapax6a* antisense probe; (**I–P**) the signals were marked by *Mapax6b* antisense probe. (**A**,**I**) 16-cell stage; (**B**,**J**) 32-cell stage; (**C**,**K**) blastula stage; (**D**,**L**) gastrula stage; (**E**,**M**) eye anlage stage; (**F**,**N**) caudal fin anlage stage; (**G**,**O**) heart-beat stage; (**H**,**P**) hatching; (**F’**,**M’**) amplification of *Mapax6a* or *Mapax6b* signal. Scale bars: 200 μm.

To further validate the expression of *Mapax6a* and *Mapax6b* during embryogenesis, in situ hybridization (ISH) on the whole mount (WISH) was then performed. Different signals were observed between the twins (Figure 4). The transcripts of *Mapax6a* were not present from the early stage to the gastrula stage (Figure 4A–D), which was similar to the detection by RT-PCR (Figure 3A). Later on, strong signals were observed in the eye anlage stage (Figure 4E), caudal fin anlage stage (Figure 4F), heart-beat stage (Figure 4G), and hatching (Figure 4H), with a prominent signal in the mid-brain compared with that in the eye (Figure 4F’). Like the expression of *Mapax6a*, the expression of *Mapax6b* was also in the early stage. However, the signal of *Mapax6b* began from the gastrula stage (Figure 4L) and was earlier than *Mapax6a* transcripts (Figure 4D), which was also confirmed by RT-PCR (Figure 3A). During the later development stages, *Mapax6a* preferred to express in the diencephalon, not in the eye (Figure 4E,F,F’), whereas *Mapax6b* was mainly detected in the eye but not in the diencephalon (Figure 4M,N,M’). Therefore, dissimilar expression patterns between *Mapax6a* and *Mapax6b* possibly suggest their different functions during embryogenesis. 

### 2.4. Mapax6a and Mapax6b Expressed in Adult Gonad besides Brain and Eye

**Figure 5:**
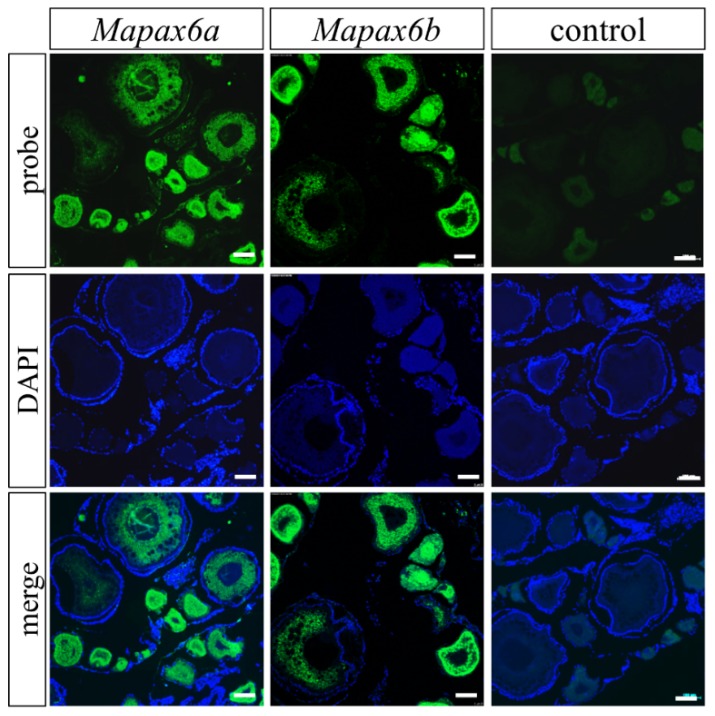
Distribution of *Mapax6a* and *Mapax6b* in the adult ovary. These sections were stained with nuclear dye 4′,6-Diamidino-2-phenylindole dihydrochloride (DAPI), represented by blue signals. The green fluorescent signal displays the intracellular distribution of *Mapax6a* and *Mapax6b* RNA in the ovary detected by antisense probe. The sense probe was used as a negative control. Merge micrographs of DAPI staining and probe staining are shown in the third row. Scale bars: 100 μm.

**Figure 6:**
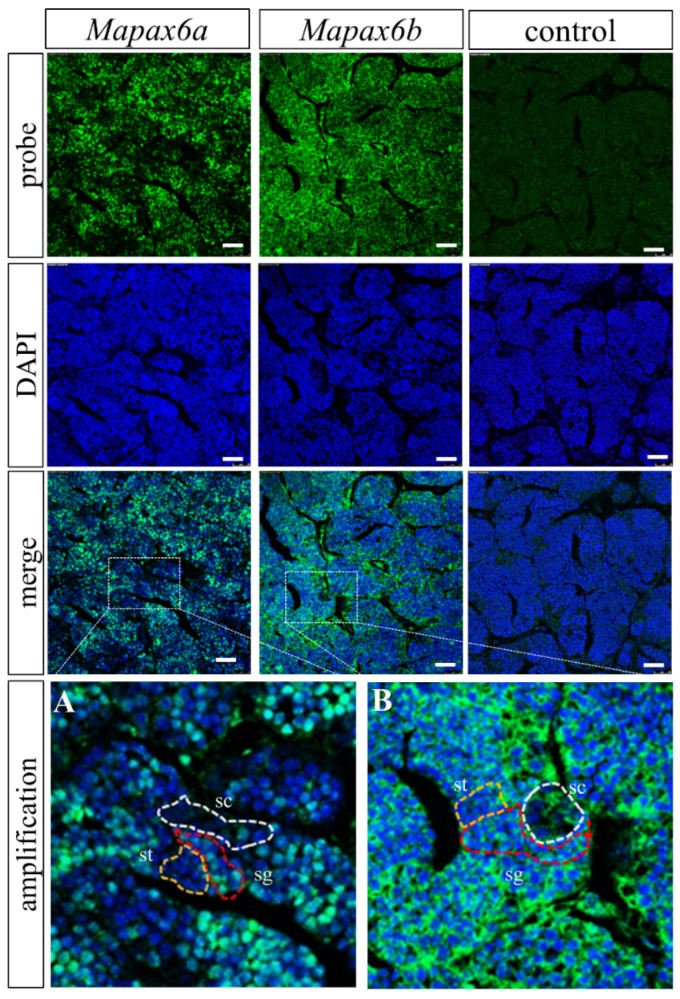
Distribution of *Mapax6a* and *Mapax6b* in the adult testis. The blue signal indicates DAPI staining, and the green signal exhibits the distribution of *Mapax6a* and *Mapax6b* RNA in the testis. The sense probe was used as a negative control. **A** and **B** reveal the amplification of the *Mapax6a* and *Mapax6b* signals, respectively. sc: spermatocytes, surrounded by white dotted lines; sg: spermatogonia, surrounded by red dotted lines; st: spermatids, surrounded by yellow dotted lines. Scale bars: 100 μm.

According to the above results, the canonic expression patterns in the brain and eyes were confirmed in *Mapax6a* and *Mapax6b*, and different signals were also present in gonads including the ovary and testis. Thus, we adopted fluorescent in situ hybridization (FISH) to investigate the expression locations of *Mapax6a* and *Mapax6b* in adult gonads. The result showed that *Mapax6a* and *Mapax6b* were expressed in the gonad and limited to germ cells (Figure 5 and Figure 6). However, the cell types that they enriched were different. In ovary sections, the signals of *Mapax6a* and *Mapax6b* were found in early oocytes (I), late pre-vitellogenesis (II), and oocytes stage III and IV. Following oocytes growing to bigger size, the signal became weak (Figure 5). In testis sections, the signal of *Mapax6a* was highly enriched in spermatogonia (sg) and weakly in spermatocytes (sc) and spermatids (st) (Figure 6A). Similarly, the signal of *Mapax6b* was found in the three cell types, and the signal was seemingly stronger in spermatogonia and spermatocytes (Figure 6B). Thus, both *Mapax6a* and *Mapax6b* are expressed in gonadal germ cells but not in somatic cells. 

## 3. Discussion

Although the *Pax6* gene was isolated almost thirty years ago, much of the focus of this broad topic has been on its roles in the central neural system [4]. In the present study, we report that the *Megalobrama amblycephala* genome contains two *pax6* genes, *Mapax6a* and *Mapax6b*. For the first time, gonadal but not maternal expression patterns with remarkably divergent expression details during embryogenesis and gametogenesis were acquired for both *Mapax6a* and *Mapax6b*. Due to high sequence identity with the Pax6 proteins of teleosts and other vertebrates, Mapax6a and Mapax6b were therefore identified as pax6 orthologs. Also, the gonadal acquisition of *Mapax6a* and *Mapax6b* suggests that duplicated *pax6s* are possibly involved in gametogenesis.

Firstly, duplicated *pax6* genes were identified in this study. Genetic duplication is ubiquitous in the evolution of organisms and is thought to be one of the most important factors for evolution. For instance, *Drosophila* contains two *pax6* homologs, *toy* and *eye*, due to gene duplication [29]. In zebrafish, *Drpax6a* and *Drpax6b* have been identified previously [7,18,19,30]. Similar to zebrafish, the *Megalobrama amblycephala* genome also contains two *pax6* genes, and they have been classified into *Drpax6a* and *Drpax6b* of zebrafish, respectively, by phylogenetic tree. All of the pax6 homologs contain PD, HD, and PST-rich transactivation domains in various species [5,6,17,18,31]. The proteins of Mapax6a and Mapax6b both contain an exon 5a in PD, which presents in other Pax6 and alters the DNA binding activity of PD [17]. A previous study revealed that *pax6* mainly has three isoforms: *pax6*, *pax6(5a)*, and *pax6∆PD*. RNase protection assay showed that the expression level of *pax6* was greater than that of *pax6(5a)* in neurogenesis [32]. Overexpression of *Pax6(5a)* promoted embryonic stem (ES) cells to differentiate into neurons [33], while deletion of exon 5a induced iris hypoplasia in mice [34]. Besides this, exon 3 was untranslated, including in the transcripts; the initiation of the ATG starts from exon 4, while the exclusion of exon 3 resulted in the initiation of the ATG from exon 2 in fish pax6. Here, more isoforms raised by alternative splicing of exon 2, exon 3, or exon 5a were also identified by sequencing, suggesting the potential function for these isoforms. In addition, similar to the zebrafish *pax6b* gene, *Mapax6b* lacks exon 4a through genomic alignment. However, the protein isoforms of pax6 including or excluding exon 4a still produce the PD, HD, and PST domains. Although *pax6b* lacks exon 4a in blunt snout bream, the conservation of gene structures and identical protein domains imply that *Mapax6a* and *Mapax6b* are possibly conserved in their function. Thus, duplicated *pax6* were identified in two chromosomes with synteny conservation in blunt snout bream. 

Secondly, expression of *Mapax6b* was earlier than that of *Mapax6a* during embryogenesis. The expression pattern of *pax6* was already reported in embryogenesis and adult tissues [5,6,7,18,35]. In the developing embryos, the expression of medaka *pax6b* (Ol*pax6.1*) was detected from the gastrula stage [17,20], which is similar to the expression of *Mapax6b*. Later than *Mapax6b*, the expression of zebrafish *pax6* began from early neurulation [19,30]. However, the expression of *Mapax6b* was in the blastula stage, earlier than that of *Mapax6a*, which was expressed in the later neural stage. Analogously, transcripts of the *Drosophila toy* gene were first detected at the cellular blastoderm stage, and the other homolog gene *ey* was found in late germband extension [29]. In addition to the divergent commencement of expression, the RNA transcripts of *Mapax6a* were located in the brain and eye at the eye anlage stage and more concentrated in the diencephalon during later development. In contrast, *Mapax6b* showed a considerable difference at the same developmental stage. *Mapax6b* was detected in both eye and diencephalon, but the primary signals were in the eye structure. Compared with the divergent expression of duplicated *pax6*, *Xenopus pax6.2* was not expressed in the lens or brain, although *pax6.1* was expressed in the eyes, brain, and pancreas [36,37].

Similarly, in zebrafish, *pax6a* and *pax6b* acquired eye and brain expression at the same stage, in which *pax6a* was detected in the telencephalon, diencephalon, and eye, whereas *pax6b* was predominantly presented in the eye, hindbrain, and pancreas but with weak expression in the brain [19]. In medaka fish, *pax6.1* (*pax6b*) was initiated from the gastrula stage [17], while *pax6.2* (*pax6a*) was maternally present (Pan et al., unpublished). In addition, divergent expression in brain and optic vesicle was also mentioned during medaka developmental stages: *pax6.1* was present in the brain and eye structure, and *pax6.2* was degenerated from optic vesicles and concentrated into the diencephalon (Pan et al., unpublished; [20]). Overall, the expression patterns of duplicated *pax6* in different species are obviously different, and expression of *pax6b (pax6.1)* or its ortholog in mammals is mainly conserved, whereas *pax6a* (*pax6.2*) acquired different regions and stages; this suggests that there are possible functional differences during embryogenesis.

Thirdly, the canonical expression of *pax6* such as in the brain, eye, and pancreas has been verified in adult tissues from invertebrates and vertebrates [6,7,17,20,29,30,37,38,39]. Among the duplicated *pax6* genes, expression of *pax6b* (*pax6.1)* was always detected in brain, eye, and pancreas, whereas expression in the pancreas was only detected from zebrafish *pax6b* [19,21], *Xenopus pax6.1* [37], medaka *pax6.1* and *pax6.2* ([17], Pan et al., unpublished), and elephant shark *pax6.1* [20], suggesting that *pax6b (pax6.1)* is still a dominant player and *pax6a (pax6.2)* may play a subfunctionalization role in the pancreas.

Finally, and unexpectedly, we detected the signals of *Mapax6a* and *Mapax6b* in gonad by RT-PCR, ISH, and Western blot, and both *pax6* showed expression in different germ cells. In the ovary, *Mapax6a* and *Mapax6b* were expressed in various stages of oogenesis. In testis, *Mapax6a* and *Mapax6b* were also detected in spermatogenesis, except for *Mapax6a* in the spermatids. Besides this, the signal of *Mapax6b* was stronger than that of *Mapax6a* in testis, which was confirmed by quantitative real-time PCR (qRT-PCR). These results suggest a novel role of *pax6* in the spermatogenesis and oogenesis of teleosts. A similar situation has been found in mouse, medaka, *Dicyema*, and *Caenorhabdits elegans*. For example, expression of *Pax6* is located in germ cells in mouse adult testis, such as in spermatogonia, spermatocytes, and round spermatids [13]. Most importantly, *Pax6* localizes at the XY body during meiotic prophase I, which suggests it may have functions in the inactivation of sex chromosomes during meiosis [13]. Moreover, in *Dicyema*, *pax6* was expressed in the hermaphroditic gonad [12], and *pax6* is related to the migration of gonad cells in *Caenorhabdits elegans* [14,40]. Our lab data (Pan et al., unpublished) also show that medaka *pax6a* (*pax6.2*), not *pax6b* (*pax6.1*), was expressed in ovary and testis. However, there has been no report about the gonadal expression pattern of *pax6* in species like zebrafish [19,21], elephant shark [20], and *Xenopus* [36,41,42]. In addition, the expression of *Mapax6a* and *Mapax6b* was initiated from a later stage—the blastula stage or neural stage—not from an early stage such as the 2-cell stage, indicating that the duplicated *pax6* acquired gonadal but not maternal expression. Thus, these results indicate a novel role of *pax6* in the spermatogenesis and oogenesis of blunt snout bream.

All these results taken together, we have demonstrated that *Mapax6a* and *Mapax6b* are the homologs of mammalian *pax6*, and the twins exhibit considerable divergence by synteny conservation, phylogenetic tree, and spatial–temporal expression pattern. Most importantly, these results enrich the expression pattern and evolutionary relationship of *pax6* by indicating that *pax6* may be involved in gametogenesis. 

## 4. Materials and Methods 

### 4.1. Fish and Embryo

A batch of adult individuals and embryos of blunt snout bream were obtained from the Ezhou breeding base of Huazhong Agricultural University (HZAU), and the healthy adult fish (500 ± 25 g) were acclimatized for 1 week before experimental manipulation. Various tissues including brain, eye, heart, kidney, liver, spleen, testis, and ovary were isolated from fish anesthetized with 100 mg/L MS-222. The embryos were collected according to the stages of embryogenesis [43,44]. All the tissues and embryos were separated into three groups for the expression analysis of RNA and protein. The first group was incubated with 1 mL TRIzol^®^ Reagent (Thermo Fisher Scientific, Waltham, MA, USA) per 50 mg tissue or 50 embryos for RNA extraction. The second group was placed at 4 °C after fixing with 4% Paraformaldehyde for in situ hybridization (ISH). The third group was quick-frozen in liquid nitrogen for Western blot analysis. All procedures complied with the protocol approved the Scientific Ethics Committee of Huazhong Agricultural University with the permit number HZAUFI-2015-005.

### 4.2. Isolation of RNA and Sequencing of cDNA 

Total RNA was extracted from tissues or embryos by using the TRIzol reagent according to a previous protocol [27]. The RNA qualities were checked using 1% agarose gel and NanoDrop 2000 (Thermo Fisher Scientific, Waltham, Mass USA). The first-strand cDNA was synthesized by using a PrimeScript^TM^ RT reagent kit with gDNA Eraser (Takara Bio, Shiga, Japan). The potential mRNA sequences of *Mapax6a* and *Mapax6b* were identified from an unpublished transcriptome database of blunt snout bream, and respective gene-specific primers were designed for amplifying *Mapax6a* (pax6a-F and pax6a-R, Table 1) and *Mapax6b* (pax6b-F and pax6b-R, Table 1). The PCR mixture included LA Taq^®^ (Takara Bio, Shiga, Japan), ovary cDNA, and primers. PCR was run in a 20 µL reaction mixture for 30 cycles (94 °C for 20 s, 60 °C for 30 s, and 72 °C for 1 min), and PCR products were cloned into the pMD18-T vector (Takara Bio, Shiga, Japan) and sequenced (http://www.tsingke.net/shop/). These sequences were used to design gene-specific primers and nest gene-specific primers for the 3′ untranslated region (UTR) amplification of *Mapax6a* and *Mapax6b*, respectively (Table 1). The rapid amplification of cDNA ends (RACE) cDNA Amplification Kit (Takara Bio, Shiga, Japan) was used to synthesize the cDNA library. The 3′RACE PCR was performed for 3 min at 95 °C, followed by 5 cycles of 95 °C for 20 s, 64 °C for 30 s, and 72 °C for 2 min; 10 cycles of annealing at 62 °C; and 20 cycles of annealing at 60 °C and then 72 °C for 10 min. The amplification products were ligated to the pMD18-T vector for sequencing. The sequences assembly of the ORF and 3′UTR was performed using DNAMAN software (https://www.lynnon.com/).

### 4.3. Bioinformatic Analyses

The protein sequences of Mapax6a and Mapax6b were predicted using DNAMAN software (https://www.lynnon.com/). Other species’ protein sequences were retrieved from the NCBI database (https://www.ncbi.nlm.nih.gov/). Then, an alignment program was performed using Vector NTI 11 (Invitrogen, Carlsbad, CA, USA) for these protein sequences. MEGA 6.0 (https://www.megasoftware.net/) with the neighbor-joining method was used to construct the phylogenetic tree. The parameter of bootstrap replications was set to 1000. To compare the synteny between the fish genes and humans, we retrieved the related genes from the NCBI database. 

### 4.4. Semi-Quantitative Reverse Transcription Polymerase Chain Reaction (sqRT-PCR) 

sqRT-PCR was used to determine the expression patterns of *Mapax6a* and *Mapax6b* in adult tissues and developing embryos. Two pairs of primers, pax6a-F/pax6a-R and pax6b-F/pax6b-R (Table 1), were applied in the PCR system running 30 cycles for *Mapax6a* and *Mapax6b*, respectively. *β-actin* (Accession number: AY170122.2) was used as the internal control with primers of β-actin qF and β-actin qR (Table 1) for amplification, and the PCR conditions were as follows: 25 cycles of 95 °C for 30 s, annealing at 60 °C for 30 s, and elongation at 72 °C for 10 s. The PCR products were separated on 1% agarose gel. 

### 4.5. Quantitative Real-Time Polymerase Chain Reaction (qRT-PCR)

An aliquot of 1 µL (10 ng/µL) cDNA template was used for real-time PCR analyses. Triplicate samples were tested using the CFX96 Real-Time PCR Detection System (Bio-Rad, Hercules, CA, USA) in a volume of 25 µL containing cDNA, primers, and SYBR^®^ Premix DimerEraser^TM^ (Takara). PCR was run for 40 cycles (95 °C for 10 s, 60 °C for 10 s, 72 °C for 10 s). The primers for *Mapax6a* and *Mapax6b* were pax6a-qF/pax6a-qR and pax6b-qF/pax6b-qR (Table 1), while *β-actin* was used as an internal control with β-actin qF/β-actin qR (Table 1). The relative expression of *Mapax6a* and *Mapax6b* in the samples was analyzed by using the 2^−∆∆*C*t^ method [45,46].

### 4.6. Western Blot Analysis

The protein of adult tissues was extracted by using a Tissue Protein Extraction Kit and quantified with the BCA Protein Assay Kit (CWBIO, Beijing, China). Protein lysates (30 µg/lane) were run on a 12% SDS-PAGE gel (Bio-Rad, Hercules, CA, USA) after being mixed with 5 × SDS-PAGE loading buffer (CWBIO, Beijing, China), then transferred to a PVDF membrane. The membrane was blocked with 5% non-fat dry milk in Tris-Buffered Saline Tween-20 (TBST) for 1 h, then incubated with anti-Pax6 rabbit polyclonal antibody (Boster, Pleasanton, CA, USA, dilution 1:250) or actin antibody (Vazyme, Nanjing, China, dilution 1:1000) overnight at 4 °C. Then, the membrane was washed three times for 15 min with TBST and incubated with HRP-conjugated anti-rabbit goat IgG secondary antibody (Vazyme, Nangjing, China, diluted 1:10,000) for 1 h at 25 °C. After the membrane was washed three times for 15 min with TBST, the ECL substrate (Bio-Rad) was used for detecting the signal of pax6 with Amersham Imager 600 (GE, Wauwatosa, WI, USA). 

### 4.7. In Situ Hybridization 

In situ hybridization (ISH) on the whole mount (WISH) and fluorescent in situ hybridization (FISH) on a section were performed as described with minor modifications [17,27]. The 3′UTRs of *Mapax6a* and *Mapax6b* PCR products were used as a probe template. Sense and antisense digoxigenin-labeled RNA probes were generated by using in vitro transcription with a digoxigenin (DIG) RNA labeling kit (Roche, Basel, Switzerland). For the WISH, the probe concentration was 1 ng/µL and the second antibody was anti-DIG antibody-conjugated alkaline phosphatase (Roche, Basel, Switzerland); finally, the hybridization signal was stained using nitroblue tetrazolium/5-bromo-4 chloro-3-indolyl phosphate (Sigma, St. Louis, MI, USA). For the FISH, the concentration of the probe was 5 ng/µL, and the anti-DIG antibody-conjugated peroxidase (Roche, Basel, Switzerland) was applied as the second antibody; then, the green hybridization signal was found by using tyramide signal amplification (TSA) Plus Fluorescein Solution (PerkinElmer, Waltham, Massachusetts, USA) staining, and the blue signal was stained by 4′,6-diamidino-2-phenylindole (DAPI, Sigma, St. Louis, MI, USA). The ISH *nanog* sense probe was used as a negative control [27]. FISH on section was imaged by TCS SP8 confocal microscopy (Leica, Wetzlar, Germany), and WISH was observed using a Leica M205 FA stereomicroscope (Leica, Wetzlar, Germany).

## Figures and Tables

**Table 1:** Primers list.

Primer Name	Sequence (5′ to 3′)	Temp (°C)	Usage
pax6b-F	ATGATGCAAAACAGTCACAGCG	59.1	sqRT-PCR
pax6b-R	GTGTGGAAGTCAAAGGGCGAAG	60.8	sqRT-PCR
pax6b GSP	CTGTCCCTGTCCAAGTTCCCG	61.9	3′RACE PCR
pax6b NGSP	CTTCGCCCTTTGACTTCCACAC	60.8	3′RACE PCR and probe
pax6a GSP	TGTACCAGTCCAAGTGCCAGG	61.4	3′RACE PCR
pax6a NGSP	CCTGACGTCTCTCGGCTTCAAG	61.7	3′RACE PCR and probe
pax6a-F	CGTCCATGATGCAAAACAGTCAC	59.5	sqRT-PCR
pax6a-R	CTTGAAGCCGAGAGACGTCAGG	61.7	sqRT-PCR
pax6a qF	CCAGCCAGACCTCATCCTACTCC	62.9	qRT-PCR
pax6a qR	CTTGAAGCCGAGAGACGTCAGG	61.7	qRT-PCR
pax6b qF	GCAACCAAGCCAGACATCTTCC	61.0	qRT-PCR
pax6b qR	GTGTGGAAGTCAAAGGGCGAAG	60.8	qRT-PCR
β-actin qF	AAAATCAAGATCATCGCCCCAC	59.0	qRT-PCR
β-actin qR	TACTCCTGCTTGCTAATCCAC	59.4	qRT-PCR

## Supplementary Materials

Supplementary materials can be found at https://www.mdpi.com/1422-0067/20/7/1710/s1. Figure S1. Nucleotides and deduced amino acid sequences of duplicated pax6. Mapax6a (A) and Mapax6b (B) cDNA. Open reading frames (ORFs) are shown in uppercase letters, whereas the 5′ and 3′ untranslated regions are indicated in lower case. The amino acid sequences are displayed underneath the ORF using single capital letter codes. The start codon and stop codon are highlighted in bold. Figure S2. Comparison of the cDNA and protein sequence of Mapax6a and Mapax6b: (A) the ORF sequence alignment; (B) the 3′UTR sequence alignment; (C) the protein sequence alignment. The same sequences are highlighted in yellow, and the percentage identities of these sequences are shown behind sequences. Figure S3: Sequences of different transcript variance of *Mapax6a* and *Mapax6b*. The sequences were obtained from our transcripts database. pax6a1–pax6a8 (a1–a8); pax6b1–pax6b10 (b1–b10). Figure S4. Multiple sequence alignment of pax6 proteins. The three frames highlight the paired domain, homeodomain, and PST-rich transactivation domain. Exon 5a was inserted into the paired domain. The species’ names, accession numbers, amino acid lengths, and the percentage identities of the full-length protein in other species compared with Mapax6a are presented at the end of the alignment.

## Abbreviations

DAPI	4′,6-diamidino-2-phenylindole
FISH	fluorescent in situ hybridization
HD	homeodomain
ISH	in situ hybridization
ORF	open reading frame
PAX	Paired Box
PD	pair domain
PST	proline–serine–threonine
qRT-PCR	Quantitative Real-Time Polymerase Chain Reaction
TBST	Tris-Buffered Saline Tween
UTR	untranslated region
WAGR	Wilms tumor, Aniridia, genitourinary abnormalities, and mental retardation
WISH	whole-mount in situ hybridization
